# Spin polarization driven by molecular vibrations leads to enantioselectivity in chiral molecules

**DOI:** 10.1126/sciadv.adv5220

**Published:** 2025-10-29

**Authors:** Shinji Miwa, Tatsuya Yamamoto, Takashi Nagata, Shoya Sakamoto, Kenta Kimura, Masanobu Shiga, Weiguang Gao, Hiroshi M. Yamamoto, Keiichi Inoue, Taishi Takenobu, Takayuki Nozaki, Tatsuhiko Ohto

**Affiliations:** ^1^ The Institute for Solid State Physics, The University of Tokyo, Kashiwa, Chiba, Japan.; ^2^ Trans-scale Quantum Science Institute, The University of Tokyo, Bunkyo, Tokyo, Japan.; ^3^ National Institute of Advanced Industrial Science and Technology (AIST), Research Center for Emerging Computing Technologies, Tsukuba, Ibaraki, Japan.; ^4^ Department of Materials Science, Osaka Metropolitan University, Sakai, Osaka, Japan.; ^5^ Institute for Molecular Science, Okazaki, Aichi, Japan.; ^6^ Graduate School of Engineering, Nagoya University, Nagoya, Aichi, Japan.

## Abstract

Chirality-induced spin selectivity (CISS) encompasses phenomena such as magnetoresistance and enantiomer separation using ferromagnets. It has recently received notable attention. Despite this growing interest, the microscopic mechanisms driving CISS remain a subject of intense debate. This paper complements and extends the prevailing interpretation that attributes CISS primarily to the electric current in chiral molecules. We propose that molecular vibrations drive spin polarization in chiral molecules and play a critical role in CISS. Our results suggest that magnetic interactions between chiral molecules and ferromagnets, analogous to interlayer exchange coupling, provide a physically consistent mechanism for the observed selectivity. Specifically, we demonstrate that molecular vibrations facilitate the alignment of spin angular momentum dependent on chirality in the presence of an external magnetic field, which is a prerequisite for exchange coupling. These findings necessitate reevaluating spin dynamics, expanding its relevance beyond traditional solid-state physics to fields such as chemical reactions, molecular biology, and drug discovery.

## Teaser

Molecular vibrations drive spin polarization and induce chirality-induced spin phenomena.

## INTRODUCTION

Chirality is a pseudoscalar that changes its sign with mirror reflection and lacks mirror symmetry. This intriguing property is widely recognized and studied in diverse fields, including physics, chemistry, biology, and astronomy (*1*, *2*). The impact of chirality on chemical reaction yields is a well-established concept. The origins and roles of homochirality in biomolecules have sparked extensive debate and research. Recently, numerous phenomena related to chirality-induced spin selectivity (CISS) have been reported in the field of physical chemistry (*3*–*30*). A seminal observation in this area was the detection of photoelectrons that exhibited finite spin angular momentum upon passing through double-stranded DNA molecules in a Mott polarimeter (*3*, *4*). This discovery sparked a series of studies and reports on various CISS-related phenomena. Noteworthy phenomena include magnetoresistance in junctions involving chiral molecules and ferromagnetic electrodes (*5*, *11*, *18*, *26*), as well as the enantiomer separation using ferromagnetic substrates (*10*, *27*, *28*). These findings are intriguing from a scientific standpoint and have substantial potential for practical applications, particularly in enantioselective synthesis.

Longitudinal spin current, which is a flow of spin angular momentum that is either parallel or antiparallel to the direction of the current, exemplifies a truly chiral system (*31*, *32*). In the context of CISS-related phenomena, electrons traversing chiral materials are considered to gain orbital angular momentum due to their helical motion. Subsequently, they achieve spin angular momentum via spin-orbit interaction. This process is analogous to magnetochiral anisotropy (*33*). However, the model attributing CISS primarily to electric current (*7*, *12*, *13*) remains a subject of debate. In the linear response regime of diffusive transport, the emergence of magnetoresistance is theoretically forbidden, as noted in previous studies (*34*). Furthermore, such models violate Onsager’s reciprocity principle. While electrical transport in CISS experiments does not necessarily confirm to Onsager’s relations, particularly when the transport is not diffusive (*35*), reports of exceptionally large CISS-induced magnetoresistance in materials with relatively low spin polarization (*11*, *18*, *26*), such as Ni, which exceeds that of established spintronic systems like the CoFeB/MgO system (*36*–*38*), challenge the validity of current-induced spin polarization and its tunneling-based explanation. Regarding enantiomer separation facilitated by achiral ferromagnets (*10*, *27*, *28*), one proposed model suggests that transient currents generated during molecular adsorption on metals induce a pair of antiparallel spins in chiral molecules (*10*). The resulting spins from the CISS are believed to have a long spin relaxation time (greater than hours) (*39*). However, nonequilibrium spin accumulation in materials typically has short spin relaxation times (<100 ps) (*40*). Consequently, achieving a comprehensive and unified understanding of CISS-related phenomena remains challenging, hindering its practical application and further advancement in the field.

**Fig. 1.**
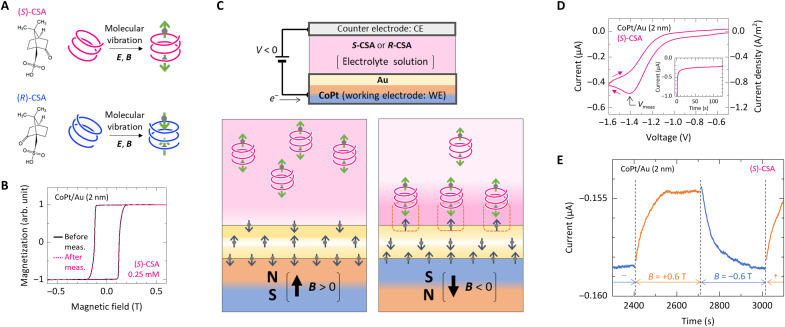
Experimental design for interlayer exchange coupling between chiral molecules and ferromagnets driven by molecular vibration. (**A**) Schematic of spin polarization driven by molecular vibration under electric (*E*) and magnetic (*B*) fields in chiral molecules, using (1*S*)-(+)-camphor-10-sulfonic acid [(*S*)-CSA] and (1*R*)-(−)-camphor-10-sulfonic acid [(*R*)-CSA]. (**B**) Magnetization hysteresis curves for the CoPt electrode, as measured by the magneto-optical Kerr effect, observed both before and after electrical measurements in an electrochemical cell, with the magnetic field oriented perpendicular to the film plane. arb. unit, arbitrary units. (**C**) A schematic illustration of the experimental setup. (**D**) Typical current-voltage characteristics of the electrochemical cell containing (*S*)-CSA at 0.25 mM and KCl at 50 mM, with a voltage sweep rate of 60 mV/s. The inset shows chronoamperometry results under a constant voltage (*V*_meas_). (**E**) Representative results of MC measurements. The working electrode had a surface area of 0.5 mm^2^. The gray and green arrows in the figure represent the spin angular momentum (*S*) and its time derivative (*dS*/*dt*), respectively.

Here, we used the prototypical chiral electrolyte (1*S*)-(+)- or (1*R*)-(−)-camphor-10-sulfonic acid (*16*) [(*S*)- or (*R*)-CSA; Fig. 1A] in a custom-made electrochemical cell with precisely engineered ferromagnetic CoPt/Au electrodes to conduct time-resolved magnetoconductance (MC) observations. We find that the essence of CISS lies in the magnetic interaction between chiral molecules and the ferromagnetic electrode, analogous to the interlayer exchange coupling (*41*). Our findings reveal a critical insight: In CISS-related phenomena, the electric current does not polarize the spins; rather, it merely probes the system. Spin alignment driven by the vibration of the chiral molecules plays a pivotal role (Fig. 1A). This vibration-driven mechanism aligns with the signature of thermally driven spin polarization (*22*, *23*) and with observations of CISS-related phenomena occurring without a bias current in the system (*14*, *15*, *21*–*23*, *42*). Furthermore, this concept is consistent with the enhancement of CISS with increasing temperature (*43*). While previous studies have noted that molecular vibration can enhance current-induced spin polarization (*44*–*46*), our work posits that molecular vibration can create a net spin angular momentum.

## RESULTS

### Experimental design and typical characteristics of the electrochemical cell

Electric measurements were carried out using a custom-made electrochemical cell (refer to Materials and Methods and fig. S1 for details). To measure the MC effect attributable to CISS, the magnetization direction of the working electrode must be aligned perpendicular to the working electrode’s film plane and the direction of the electric current. CoPt, a ferromagnetic material with perpendicular magnetization (see Fig. 1B), was chosen because of its notable corrosion resistance in electrolyte environments. This characteristic favors CoPt over the commonly used Ni in CISS-related studies. To protect the CoPt surface and to address its interaction with the electrolyte, a Au spacer layer was added to construct a CoPt/Au working electrode. Figure 1B shows the magnetization hysteresis curves of the CoPt/Au (2 nm) electrode obtained through magneto-optical Kerr effect measurements before and after evaluating the MC effect. These curves reveal no alteration in the coercive field of the CoPt electrode following electrical measurement in a 0.25 mM CSA electrolyte solution, confirming the CoPt/Au electrode’s corrosion resistance in an electrolyte environment. Figure 1C depicts the MC measurement setup using the electrochemical cell with the CoPt/Au electrode as the working electrode. The electrolyte solution consisted of H_2_O, the chiral electrolyte CSA, and the achiral supporting electrolyte potassium chloride (KCl). Our experimental design used a CSA concentration of 0.25 mM, lower than in prior studies (*16*) (20 mM), to minimize the corrosive effects of the electrolyte on the CoPt/Au electrode and ensure a well-defined interface between the CoPt/Au electrode and CSA electrolyte.

Figure 1D shows the typical current-voltage characteristics observed in our electrochemical cell. When a negative voltage was applied starting from 0 V, the electric current exhibited a peak at −1.4 V. In this configuration, the working electrode functions as an anode under the applied negative voltage, facilitating the movement of electrons from the working electrode to the electrolyte solution. The anode reaction is crucial in determining the overall electric current in the system because the surface area of the working electrode is smaller than that of the counter electrode (see fig. S1, A and B). The current in the electrochemical cell increases with the addition of CSA (see fig. S1, C and D). Hence, the electrode reaction characterized by the current peak around −1.4 V is due to the oxidation (reduction) in the CoPt/Au electrode (CSA electrolyte). Because the reduction involves CSA accepting electrons to facilitate the conversion of camphor to borneol, this electrode reaction is dominant in Fig. 1D. To evaluate the MC effect, which is the variation in current as a function of the magnetization direction of CoPt, the voltage was set at *V*_meas_. The electric current was then measured under this constant voltage setting (chronoamperometry). Notably, *V*_meas_ depends on the thickness of the Au film, showing a slight increase as the Au thickness increases (fig. S1E).

When a constant voltage (*V*_meas_) is applied to the electrochemical cell, an initial surge of relatively large current (>1 μA) is observed. This current then abruptly declines, as shown in Fig. 1D (inset). This phenomenon can be attributed to two primary factors. First, there is a reduction in the amount of ion movement in the solvent. When a constant voltage is applied, ions move through the electrolyte solution and form an electric double layer near the electrode surface. As this double layer forms, ion movement decreases, leading to a reduction in current. Second, the electrolyte concentration decreases near the electrode surface due to electrochemical reactions. Electrolyte at the electrode surface is consumed by these reactions. When the consumption exceeds the electrolyte supply from the bulk solution through diffusion, the electrolyte concentration near the electrode decreases, resulting in a decrease in current. Over time, as these processes reach a balance, the current stabilizes, primarily constrained by the diffusion rate of the electrolyte to the electrode surface. In this quasisteady state, the presence of 0.25 mM CSA influences the current, with a difference of more than a twofold observed (fig. S1D). This variation highlights that the electric current is primarily driven by the electrochemical reaction at the CoPt/Au electrode interface with CSA. The MC effect was assessed in this quasisteady condition.

Figure 1E shows typical results demonstrating the MC effect. A constant magnetic field of 0.6 T was applied perpendicular to the electrode surface using an electromagnet. The polarity of the magnetic field was alternated every 300 s, with each transition taking ~5 s. The data show that switching the polarity of the magnetic field from negative to positive (and vice versa) leads to a gradual decrease (or increase) in current until a steady state is established, with a relaxation time of about 50 s. The MC effect diverges from predictions made by traditional theories, such as spin-dependent tunneling similar to tunneling magnetoresistance effect (*36*–*38*, *47*). If changes in interface conductance due to spin-dependent tunneling were responsible, then we would expect an immediate response in current following a change in the polarity of the magnetic field, with relaxation trends counteracting this change. However, our experimental results indicate a monotonous decrease or increase in conductance following a change in the polarity of the magnetic field. This suggests that a mechanism other than spin-dependent tunneling conduction is required to explain the MC effect. The electric current in the steady state is governed by physical parameters related to the bulk electrolyte concentration and diffusion coefficient (*48*). Because it is unlikely that the concentration of the bulk electrolyte depends on the magnetization direction, we propose that the diffusion coefficient of CSA near the CoPt/Au interface depends on the magnetization direction. This variation alters the CSA supply rate to the electrode surface, thereby affecting the local CSA concentration and, consequently, the electric current. A deeper analysis of this phenomenon and its implications will be provided in subsequent discussions.

### Time-resolved MC measurements

**Fig. 2.**
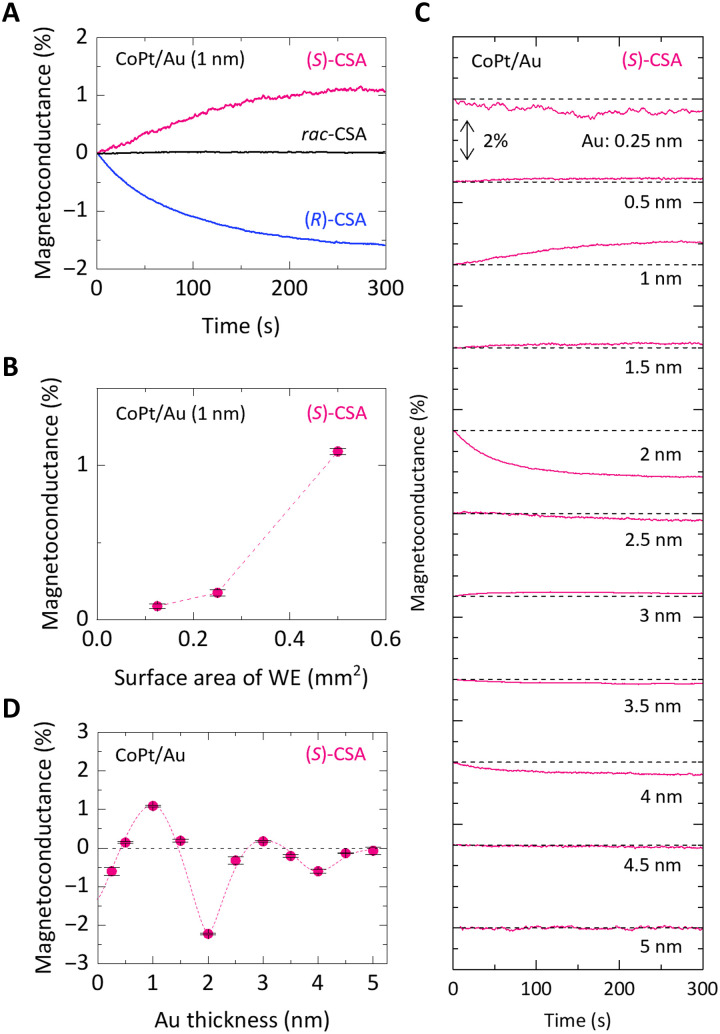
Time-resolved MC measurements. (**A**) Chirality dependence on the MC effect using a CoPt/Au (1 nm) electrode. (**B**) Variation of the MC ratio with surface area of the working electrode (WE). (**C**) Influence of Au thickness on the MC effect. (**D**) The MC ratio as a function of Au spacer thickness. For all data shown in the figure, the concentrations of CSA and KCl are set to 0.25 and 50 mM, respectively. The dashed curve is a guide to the eye. Except for (B), the surface area of the working electrode is fixed at 0.5 mm^2^.

Figure 2 explores the chirality dependence of CSA, the impact of working electrode size, and the effect of the thickness of the Au spacer layer to elucidate the mechanism behind the MC effect. Figure 2A shows the changes in conductance over 300 s after switching the polarity of the magnetic field, using a CoPt/Au (1 nm) electrode setup similar to that described in Fig. 1E. The MC effect was defined as (*I*_*B* = +0.6 T_ − *I*_*B* = −0.6 T_)/(*I*_*B* = +0.6 T_ + *I*_*B* = −0.6 T_). Notably, the experiment revealed that (*S*)-CSA induced a positive MC effect, whereas (*R*)-CSA resulted in a negative MC effect. In trials using a racemic mixture of CSA, the MC effect was observed to be minimal. These results are consistent with CISS-related phenomena reported in previous studies, highlighting the critical role of molecular chirality in determining the polarity of the MC effect.

Figure 2B shows how the MC ratio varies with the surface area of the working electrode. Notably, the MC effect was minimal (<0.1%) for a small electrode with an area of ~0.1 mm^2^. However, a MC effect (>1% at 0.5 mm^2^) was observed as the electrode size increased to 0.5 mm^2^. This tendency of the MC effect with respect to the electrode area challenges the notion that its origin lies in spin-dependent tunneling conduction because the MC effect is not expected to vary with electrode size under this mechanism. The key factor influencing the electrode reaction rate with changes in electrode size is alternation in electrolyte diffusion. For larger electrodes that exceed the thickness of the diffusion layer (Gouy-Chapman layer), i.e., the area near the electrode where the electrolyte concentration diverges from the bulk concentration, electrolyte diffusion tends toward a one-dimensional model perpendicular to the electrode surface. Conversely, for smaller electrodes that do not meet this criterion, the edge effect becomes substantial. This leads to three-dimensional diffusion and an enhanced rate of electrolyte supply to the electrode interface. The increased supply with the smaller electrode sizes could explain the observed reduction in the MC effect. Thus, the experiment posits that the MC effect originates from magnetic interactions tied to the magnetization direction of the CoPt/Au electrode and the chirality of the CSA electrolyte, which influences the CSA concentration near the electrode surface. This alteration in concentration occurs before the electrochemical reaction, i.e., the electron transfer process from the CoPt/Au electrode to CSA electrolyte. This suggests the presence of a magnetic interaction between CoPt/Au and CSA that is independent of electric current flow. Understanding the nature and origin of this magnetic interaction is essential for comprehensively understanding the MC effect. For all the measurements, other than Fig. 2B, a working electrode with an area of 0.5 mm^2^ was used.

To elucidate the nature of the magnetic interaction underlying the MC effect, we explored how it varies with the thickness of the Au spacer layer. Figure 2C shows the characteristics of the MC effect’s dependence on Au thickness. The corresponding MC ratio is shown in Fig. 2D. Figure 2D reveals that the MC ratio begins with a negative value at nearly zero Au thickness and oscillates with a period of ~2 nm, including reversals (see also fig. S4). This oscillatory behavior is analogous to the interlayer exchange coupling observed in Fe/Cr/Fe and Fe/Au/Fe systems, which show similar oscillation periods (*41*, *49*, *50*). Such coupling is usually attributed to conduction electrons mediating interactions via Ruderman-Kittel-Yosida mechanism (*51*–*53*), or it may arise from surface magnetic reconstruction (*54*). In either case, the trend observed in Fig. 2D strongly suggests the presence of an oscillatory exchange coupling between CoPt and CSA. This suggests that the chirality-dependent spin polarization of CSA plays a pivotal role in the manifestation of the MC effect, independent of electric current flow. As mentioned previously, the MC effect observed in Fig. 2 occurs at a CSA concentration of 0.25 mM. This effect remains almost unchanged with increased CSA concentrations. A comparable MC effect was noted at concentrations up to 4 mM (see fig. S2, A and B).

In measuring the time-resolved MC effect, we first saturate the magnetization of the CoPt electrodes by applying a magnetic field of +0.6 T (−0.6 T) and then maintain this field strength throughout the measurement process. CoPt is magnetized perpendicular to the plane of the electrodes. The magnetization at zero magnetic field after saturation at ±0.6 T is nearly identical to the magnetization at ±0.6 T. Thus, if the role of external magnetic field is solely changing the magnetization direction of the CoPt electrodes, then the MC effect would be observed regardless of whether the magnetic field is applied during the measurements. However, when we saturate the magnetization of the CoPt electrodes with a magnetic field of ±0.6 T and then conduct a measurement at a zero magnetic field, no MC effect is observed (see fig. S2B). Thus, the absence of the MC effect in zero-field measurements strongly suggests that the external magnetic field contributes to both the magnetization reversal in CoPt and the induction of chirality-dependent spin polarization in CSA molecules.

The MC ratio depicted in Fig. 2 peaks at ~3%, which is lower than the ~20% MC effect reported in previous studies that used conventional cyclic voltammetry cell with Ni/Au (10 nm) electrodes and a higher concentration of CSA (20 mM) (*16*). This discrepancy prompted an investigation into the effect of CSA concentration on the MC effect. For instance, using a 0.25 mM CSA solution with a CoPt/Au (1 nm) electrode setup produced a +1.1% MC effect, as shown in Fig. 2A. In contrast, using an 8 mM CSA solution generated a −3.2% MC effect, effectively tripling the MC ratio, as depicted in fig. S2 (C and D). However, unlike the stable magnetization curves of CoPt observed with CSA concentrations below 4 mM, an alteration in the hysteresis curve was detected following measurements at 8 mM CSA (see fig. S2C). This suggests that a high concentration of CSA can induce a direct reaction between CSA and CoPt, leading to CoPt dissolution into the solution despite a Au layer covering the electrode surface. The shift in the MC effect’s polarity from positive at 0.25 mM to negative at 8 mM in the CoPt/Au (1 nm) electrodes indicates a direct electrochemical reaction between CSA and CoPt that is unaffected by the Au layer. The relatively modest MC effect in Fig. 2 may be due to the low CSA concentration used and the selection of a ferromagnetic electrode (CoPt), which is known for its corrosion resistance and helps maintain a well-defined metal/solution interface. Using a more soluble electrode, such as Ni, or a higher electrolyte concentration appears to enhance the MC effect while compromising the integrity of the interface.

### Theoretical studies on spin polarization in chiral molecules

Inducing the MC effect requires establishing a net spin angular momentum in CSA molecules without an electric current. Previous studies have shown that molecular vibrations can enhance current-induced spin polarization (*44*–*46*), but they have not demonstrated the generation of net spin angular momentum without an electric current. However, our experiments provide compelling evidence that external magnetic fields contribute to the induction of chirality-dependent spin alignment in molecules. Accordingly, we explored how molecular vibrations under a magnetic field influence the spin polarization of chiral molecules using first-principles calculations (see Materials and Methods).

**Fig. 3.**
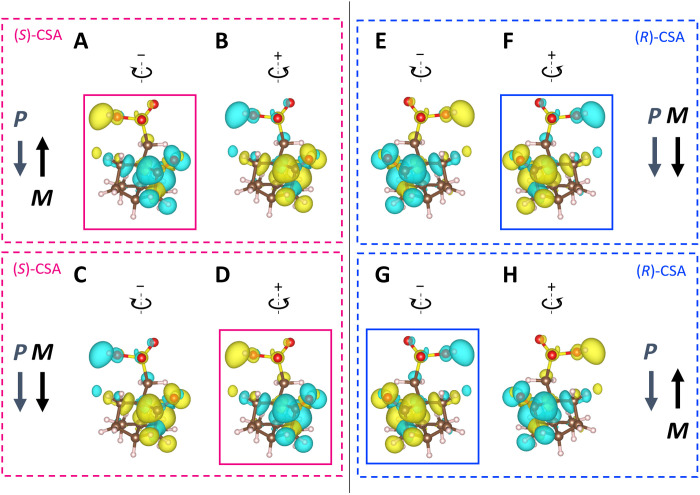
First-principles study. (**A** and **B**) Schematic diagrams of the computational model for (*S*)-CSA showing spin density under antiparallel electric polarization (*P*) and magnetization (*M*) configurations. The contours indicate changes in spin angular momentum (*dS*/*dt*) for positive (+) and negative (−) displacements. Yellow indicates *dS*/*dt* > 0 (upward), and cyan indicates *dS*/*dt* < 0 (downward). This model identifies a key molecular vibrational mode at 1770 cm^−1^. (**C** and **D**) Identical calculations for (*S*)-CSA, but with the parallel alignment of *P* and *M*. (**E** to **H**) Similar analyses for (*R*)-CSA. Considering the vibrational angular momentum and spin-vibration coupling in a magnetic field, molecular vibrations can cause variations in polarity duration where *dS*/*dt* > 0 and *dS*/*dt* < 0. This leads to a predominance of *dS*/*dt* density in (A), (D), (F), and (G), as indicated by the solid squares.

To induce a finite spin density in the closed-shell CSA molecule, which has no intrinsic spin density, we introduced 0.1 electrons into its molecular structure. As depicted in Fig. 3 (A and B), the results show the spin density difference isosurfaces with a value of ±0.0001μ_B_ Å^−3^ for (*S*)-CSA post–energy relaxation, with downward electric polarization and upward (downward) magnetization (spin angular momentum). The figure also highlights a molecular vibrational mode at 1770 cm^−1^, which is characterized by substantial vibrational circular dichroism (VCD) and the largest electron-vibration coupling constant (see fig. S3). It shows changes in spin angular momentum (*S*) with positive (+) and negative (−) displacements, yellow for *S* > 0 (upward) and cyan for *S* < 0 (downward). Displacements are defined as positive (negative) when they lead to a decrease (increase) in the electric polarization of the CSA molecule. With respect to the carbon and oxygen atoms of the CSA molecule, which exhibit substantial changes in spin density, these positive (negative) displacements align with clockwise (anticlockwise) movements. Because molecular vibrations involve alternating displacements in both directions, the resulting contours can be interpreted as the time derivative of the spin angular momentum (*dS*/*dt*). Calculations for reversed polarization directions, as well as for the (*R*)-CSA, are presented in Fig. 3 (C to H).

Initially focusing on Fig. 3 (A to D), we notice that the spin densities exhibit opposite polarities in response to + and − displacements. However, because the half-periods, τ_+_ and τ_−_, are identical, it is unlikely that the CoPt/Au ferromagnetic electrode will interact with the CSA in a manner influenced by the electrode’s spin polarization. This is consistent with the principle that chirality is time-reversal even and does not facilitate the induction of net spin angular momentum in an equilibrium state (*31*, *32*).

It is known that magnetic fields can alter the period of circularly polarized phonons (*55*, *56*). Considering the molecular vibrations, we expected differences in the half-periods τ_+_ and τ_−_ due to the magnetic field’s influence. This is corroborated by the spin-vibration coupling (*56*) and vibrational angular momentum. For instance, in Fig. 3 (A and B), we predict that τ_−_ will be greater than τ_+_ and the opposite will be true in Fig. 3 (C and D). Despite the time integral of *dS*/*dt* remaining zero, molecular vibrations introduce a disparity in durations exhibiting polarities *dS*/*dt* > 0 and *dS*/*dt* < 0 in (*S*)-CSA. This leads to dominance in the *dS*/*dt* density of Fig. 3 (A and D compared to B and C), as indicated by the pink solid squares. Similarly, for (*R*)-CSA, (F) and (G) of Fig. 3 are more pronounced than (E) and (H), as shown by blue solid squares. Thus, the application of a magnetic field induces time variance in *dS*/*dt* polarities based on chirality, triggering the CISS effect. As CSA molecules approach the CoPt/Au electrode, the electrode responds such that *dS*/*dt* < 0 in a positive magnetic field and *dS*/*dt* > 0 in a negative one. Therefore, the interaction between the spin polarizations (*dS*/*dt*) of the CSA and the ferromagnetic electrode is influenced by chirality and the direction of the ferromagnetic electrode’s spin polarization, thereby generating a measurable MC effect.

Exploring how the CISS effect depends on magnetic field strength and temperature is intriguing. According to the above theory, an external magnetic field is essential for creating spin-density asymmetry in molecular vibrations or the difference in oscillation periods between (A) and (B) of Fig. 3. Consequently, the spin density increases monotonously with the external magnetic field until the magnetic moments of the chiral molecules are fully polarized. We have confirmed experimentally this relationship between the MC effect and external magnetic field strength up to 0.6 T (see fig. S5). Regarding temperature dependence, the amplitude of molecular vibrations is expected to increase as the temperature rises from absolute zero, leading to a monotonic increase in spin density (see Supplementary Text and fig. S6).

## DISCUSSION

**Fig. 4.**
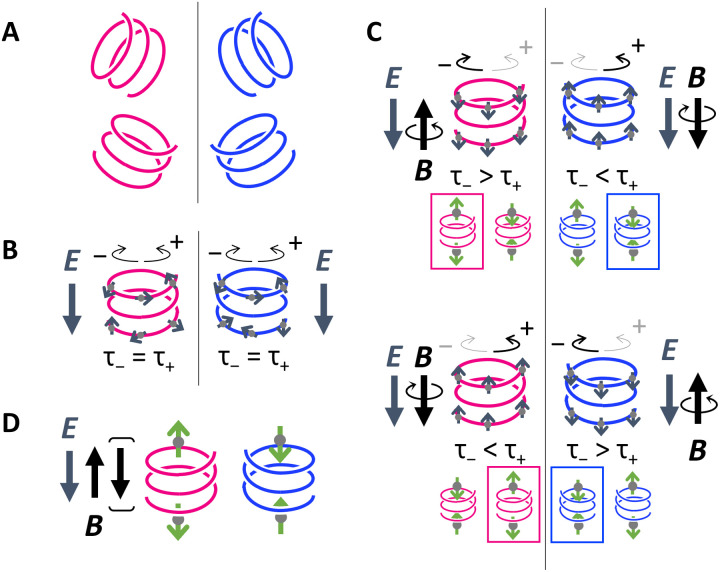
Spin density in chiral molecules driven by vibration. (**A** and **B**) Chiral molecules align under an electric field (*E*), which orients their molecular structures. (**C**) A magnetic field (*B*) results in a chirality-independent spin density alignment. At finite temperatures, the interplay between electron-vibration coupling and spin-orbit interaction can result in chirality-dependent spin density at the edges of the chiral molecules. Spin-vibration coupling under a magnetic field is expected to cause variations in the durations of half-periods τ_+_ and τ_−_, due to vibrational angular momentum. Gray and green arrows represent the spin density (*S*) and its time derivative (*dS*/*dt*), respectively. (**D**) The time derivative of the spin density (*dS*/*dt*) in right-handed and left-handed molecules, affected by both *E* and *B*. The direction of *B* affects the polarity of spin density and the relative half-period durations. Consequently, the dominant *dS*/*dt* polarity remains consistent regardless of the direction of *B*, as it is determined solely by molecular handedness.

The concept of spin alignment driven by molecular vibration, as demonstrated in the first-principles study, is shown schematically in Fig. 4. Figure 4A shows initially freestanding chiral molecules that are randomly oriented and lack net spin angular momentum. When an electric field (*E*) is applied, the molecules align, as shown in Fig. 4B. The electric field also induces a charge, which disrupts the molecule’s closed-shell structure and generates spin density, as indicated by the gray arrows. Furthermore, applying a magnetic field (*B*) leads to a chirality-independent alignment of the spin density (*S*), as illustrated in Fig. 4C. However, this spin density does not lead to CISS because it lacks an even function component. At finite temperatures, a vibrational mode may emerge in which neighboring atoms move in parallel along a helical trajectory. This collective atomic motion shifts the uniform spin density along the helix. Consequently, no net spin density is induced in the central region. Instead, spin density occurs only at the helix edges, as indicated by the green arrows in Fig. 4C. For instance, Fig. 4C (left) shows that atomic motion in the positive direction results in spin-down accumulations at the top edge and depletion at the bottom edge, producing opposite spin density at the two edges. The green arrows denote the time derivative of the spin density (*dS*/*dt*), which changes sign with clockwise (+) and counterclockwise (−) vibrations. Due to vibrational angular momentum and spin-vibration coupling in an external magnetic field, the half-periods of these vibrations differ. This leads to spin density polarities (*dS*/*dt* > 0 or *dS*/*dt* < 0) driven by molecular vibrations as shown in Fig. 4D. The magnetic field influences both the spin density polarity and the relative half-period durations. Spin density is independent of magnetic field direction and dependent solely on molecular handedness.

Several key aspects emerge when comparing CISS-related phenomena with the findings of this study. The spin polarization of photoelectrons traversing chiral molecules (*3*, *4*) and the chirality-dependent circular photogalvanic effect (*20*), which are attributed to current-induced bulk spin polarization, are outside the scope of this discussion due to symmetry considerations. The use of achiral ferromagnetic metals for enantiomer separation (*10*, *27*, *28*) appears to align with the proposed mechanism in this research. While previous studies have discussed transient current-induced spin polarization during molecule adsorption (*10*) or under electric fields (*17*), short spin relaxation times within materials (<100 ps) (*40*) make this unlikely in practical scenarios. This study proposes that exchange coupling driven by molecular vibration is substantial in achieving enantiomer separation with ferromagnetic electrodes. The underlying mechanisms of spin polarization in chiral molecules, responsible for bias current–free CISS phenomena (*14*, *15*, *21*–*23*), have remained elusive thus far. However, spin alignment driven by molecular vibration provides a clear explanation. Regarding the effective field observed through magnetization switching (*9*) and skyrmion motion (*30*), we believe that its intrinsic origin lies in the magnetic interaction proposed in this study. However, interpreting both the length scale and magnitude of this interaction requires further consideration (see Supplementary Text). Regarding the magnitude of the CISS effect, theoretical studies have reported that surface spin polarization in spacer metals (e.g., Au) can enhance the CISS phenomenon (*57*). In the context of the chirality-induced exchange coupling explored in this study, in addition to the straightforward scenario in which the spin of chiral CSA molecules directly interacts with the spin of ferromagnetic CoPt via exchange coupling, it is also plausible that the surface spin polarization of Au is modulated by the spin polarization of CSA. This induced polarization could couple with CoPt through an exchange coupling, which could potentially amplify the observed effect. Notably, such surface spin polarization has been experimentally observed on Au surfaces modified with thiol groups (*58*). These effects tend to be more pronounced in systems involving covalent bonding, such as self-assembled monolayers of thiol-functionalized molecules on Au surface. However, in physisorbed systems, such as the electrolyte solution used in this study, the induced magnetic moment at the Au surface is likely relatively small.

Additionally, previous discussions on magnetoresistance effects at the interface between chiral molecules and ferromagnetic metals have focused on electric current in molecules and spin-dependent tunneling at the interface. However, our study suggests that interlayer exchange coupling driven by molecular vibration could alter the adsorption state of chiral molecules. These changes could affect the electrical resistance at the interface due to structural adjustments. This could potentially explain the magnetoresistance effects observed, which are not solely attributable to spin-dependent tunneling. Studies using identical chiral molecules have reported exceptionally high magnetoresistance ratios (>90%) with conductive atomic force microscope setups, in stark contrast to the substantially lower magnetoresistance effects (<1%) observed in multilayer device structures (*11*, *18*). This discrepancy may be due to the dynamic nature of molecular structures in atomic microscope setups, which allows for easier structural adjustment compared to the more rigid multilayer devices. CISS-induced magnetoresistance effects in studies involving electrolytes (*8*, *16*), including this study, tend to be pronounced, possibly because the molecules are not rigidly fixed.

This study sheds light on the mechanism behind the CISS-induced magnetoresistance and enantiomer separation, which is crucial for understanding CISS-related phenomena. Our findings suggest that these phenomena originate from chirality-dependent spin alignment in molecules driven by vibration and their exchange coupling with ferromagnets. This connection extends beyond CISS to broader applications in chemical reactions, molecular biology, and drug discovery. Thus, a substantial shift in system design and analysis across these diverse fields may be necessary. This underscores the importance of a more comprehensive and integrated approach to exploring and harnessing the intricate relationship between chirality and spin dynamics.

## MATERIALS AND METHODS

### Custom-made electrochemical cell

We used a custom-made electrochemical cell, the schematic of which is presented in fig. S1A. The multilayer structure, comprising Ta (5 nm), Pt (10 nm), and [Co (0.25 nm)/Pt (0.15 nm)]_20_, was fabricated on a thermally oxidized silicon substrate using the magnetron sputtering method (*59*). After fabrication, the sample was transferred to an electron beam deposition system for further processing. First, we cleaned the sample surface with soft Ar-ion etching. Then, we deposited Co (0.25 nm) and Au (0 to 5 nm) layers under ultrahigh vacuum. The multilayer films were then patterned into electrodes suitable for the electrochemical cell using conventional photolithography techniques (with AZ6124 and LOR-3A resists) and Ar-ion etching. As depicted in fig. S1B, the working electrodes of various sizes (0.13 to 0.5 mm^2^) were prepared, each substantially smaller than the counter electrode (16 mm^2^). This design ensures that the electric current is limited by the anode reaction, i.e., the electrochemical reduction (oxidation) of the electrolyte (electrode).

After the microfabrication, the samples underwent cleaning. This included an ultrasonic bath with acetone, 2-propanol, and H_2_O, followed by a 5-min treatment in an ultraviolet (UV) cleaner (ASM401N, Asumigiken Ltd.) to remove any residual resist from the sample surface. Last, a custom-designed acrylic wall (Yumoto Electric Inc.) was fixed to the substrate using Kapton tape and UV curing resin to form the electrochemical cell as shown in fig. S1B.

### Electrolyte solution

The electrolyte solution consisted of ultrapure water [Ultrapure Water (H_2_O), Fujifilm Wako Pure Chemical Co.], a chiral electrolyte [(*S*)-CSA or (*R*)-CSA; Sigma-Aldrich], and an achiral supporting electrolyte (KCl; Fujifilm Wako Pure Chemical Co.). These reagents were used directly as purchased. In all the experiments in Figs. 1 and 2, CSA was used at a concentration of 0.25 mM, while KCl was maintained at 50 mM. A 30-μl volume of the electrolyte solution, prepared in a nitrogen glove box (UNICO Ltd.), was added to the electrochemical cell. The cell was then sealed and removed from the glove box for the subsequent electrical measurements.

### MC measurement

Electrical measurements were carried out using a two-terminal method with a custom-made probe system. The system was integrated with a Keithley 2450 source measure unit and an electromagnet (Tesla Ltd.) to characterize the MC effect. All measurements were conducted at room temperature.

### First-principles calculation

Density functional theory calculations were performed with the Vienna Ab initio Simulation Package (VASP) code (*60*) with the projected augmented wave method (*61*) and the Perdew-Burke-Ernzerhof (PBE) exchange-correlation functional (*62*). The plane-wave energy cutoff was set to 500 eV. A CSA molecule with the molecular dipole moment aligned along the *z* axis was placed in a cubic cell with an edge length of 20 Å. Because the calculation included spin-orbit coupling, noncollinear spin calculations were used. Initial magnetization was achieved by adding 0.1 electrons, setting the direction of the magnetic moment to either +*z* or −*z*. Frozen phonon calculations were performed to obtain vibrational modes.

We performed additional calculations to identify significant vibrational modes. First, we calculated the electron-vibration coupling between electronic states and vibrational modes. We used the SIESTA package (*63*) modified to compute electron-vibration coupling constants (*64*). The electron-vibration interaction Hamiltonian is

$$H_{e-\text{ph}}=\sum_{m}\sum_{i,j}M_{\mathrm{ij}}^{m}c_{i}^{†}c_{j}\left(b_{m}^{†}+b_{m}\right)$$ (1)

$$\begin{array}{c} M_{\mathrm{ij}}^{m}=\sum_{A\nu }{\left\langle i\left|\frac{\partial H_{e}}{\partial R_{A\nu }}\right|\;j\right\rangle }_{Q=0}Q_{A\nu }^{m}\sqrt{\frac{\hbar }{2M_{A}\omega_{m}}} \end{array}$$ (2)

where $c_{i}^{†}$ and $c_{j}$ are the creation and annihilation operators of an electron, respectively, and *i* is the index of the basis function. $b_{m}^{†}$ and $b_{m}$ are the creation and annihilation operators of vibration *m*, with frequency $\omega_{m}$ and eigenvector $Q_{A\nu }^{m}$ . Here, *A* and ν go over the atom and Cartesian axes *x*, *y*, and *z*, respectively. $M_{A}$ is the mass of atom *A*. $R_{A\nu }$ is the displacement around the equilibrium position and $H_{e}$ is the electron Hamiltonian. To calculate Eq. 3 practically, we rewrite it as

$$\begin{array}{c} \left\langle i\left|\frac{\partial H_{e}}{\partial R_{A\nu }}\right|\;j\right\rangle =\frac{\left\langle i\;|\partial H_{e}\;|\;j\right\rangle }{\partial R_{A\nu }}-\left\langle i^{\prime }\;|H_{e}\;|\;j\;\right\rangle -\left\langle \;i\;|H_{e}\;|\;j^{\prime }\;\right\rangle \end{array}$$ (3)

where $|\;i^{\prime }\;\rangle \;=\;\partial \;|\;i\rangle \;/\;\partial R_{A\nu }$ is the change in the basis function due to the atomic displacement. This term appears because SIESTA uses a linear combination of atomic orbitals. Using the completeness condition via overlap integral **S**

$$\sum_{\mathrm{ij}}|\;i\;\rangle {\left(S^{-1}\right)}_{\mathrm{ij}}\langle \;j\;|=1$$

we obtain

$$\begin{array}{c} \begin{array}{c} \left\langle i\left|\frac{\partial H_{e}}{\partial R_{A\nu }}\right|\;j\right\rangle =\frac{\left\langle i\;|\partial H_{e}\;|\;j\right\rangle }{\partial R_{A\nu }}-\sum_{\mathrm{kl}}\left\langle i^{\prime }\;|\;k\right\rangle {\left(S^{-1}\right)}_{\mathrm{kl}}\left\langle l^{\prime }\;|\;H_{e}\;|j\right\rangle \\ -\sum_{\mathrm{kl}}\left\langle i\;|H_{e}\;|\;k\right\rangle {\left(S^{-1}\right)}_{\mathrm{kl}}\left\langle l\;|j\right\rangle \end{array} \end{array}$$ (4)

This can be calculated by modifying the SIESTA program slightly to output the Hamiltonian and the overlap matrix elements for each coordinate. Note that Eq. 1 assumes only the linear coupling term; therefore, the Born approximation, which excludes multiple scattering interactions, is also assumed. For the later analyses, it is useful to transform the atomic basis to the molecular orbital basis. In the *I* and *J* molecular orbital basis, Eq. 2 can be rewritten as

$$\begin{array}{c} \begin{array}{c} M_{\mathrm{IJ}}^{m}=\sum_{A\nu }\left(\left\langle I\left|\frac{\partial H_{e}}{\partial R_{A\nu }}\right|J\right\rangle -\varepsilon_{J}\left\langle \left.\frac{\partial }{\partial R_{A\nu }}I\right|J\right\rangle -\varepsilon_{I}\left\langle I\left|\frac{\partial }{\partial R_{A\nu }}\right.J\right\rangle \right)\\ Q_{A\nu }^{m}\sqrt{\frac{\hbar }{2M_{A}\omega_{m}}} \end{array} \end{array}$$ (5)

where $\varepsilon_{I}$ is the energy of the molecular orbital *I*, and, when *I* is equal to *J*, the coupling is related to the molecular orbital deformation according to the normal mode vector

$$M_{\mathrm{II}}^{m}=\sum_{A\nu }\frac{\partial \varepsilon_{I}}{\partial R_{A\nu }}Q_{A\nu }^{m}\sqrt{\frac{\hbar }{2M_{A}\omega_{m}}}$$ (6)

Here, we calculated the electron-vibration coupling constant for the highest occupied molecular orbital (HOMO) and the lowest unoccupied molecular orbital (LUMO) as

$$\lambda^{m}=\lambda_{\text{HOMO}}^{m}+\lambda_{\text{LUMO}}^{m}$$ (7)

where $\lambda_{I}^{m}={\left(M_{\mathrm{II}}^{m}\right)}^{2}\;/\omega_{m}$ . For the SIESTA calculations, we used the PBE functional and the double-ζ plus polarization basis set. The cutoff for the real space grid was set to 300 rydberg.

Second, we calculated the VCD spectrum using the Gaussian 16 program (*65*) at the B3LYP/6-31G (d,p) level to study the coupling between each vibrational mode and the molecular chirality. Circular dichroism (CD) is the differential absorption of left (L) and right (R) circularly polarized light

$$∆a=a^{L}+a^{R}$$ (8)

CD can be expressed in terms of rotational strength as (*66*)

$$∆a\propto R=\omega^{-1}\frac{\partial \mu }{\partial t}⊗m$$ (9)

where μ and *m* are the electric dipole and magnetic dipole moments, respectively. Therefore, a large VCD indicates a strong interaction between the molecular vibrations and the magnetic moments. Last, we calculated the spin-vibration coupling using the VASP package, which is the change in the magnetization according to the displacement along the normal mode (~0.1 Å).

All the results are shown in table S1. We adopted the frequency calculated with VASP because the order of the vibrational modes was consistent across the three packages, despite slight differences in the frequencies. Although calculations show that every mode has finite spin-vibration coupling and VCD, we selected a representative vibrational mode, C═O stretching mode (1770 cm^−1^), as shown in fig. S3, which has the largest electron-vibration coupling constant.

## Supplementary Materials

### This PDF file includes:

Supplementary Text Figs. S1 to S6 Table S1 References
