# Supplementary material for: Spin polarization driven by molecular vibrations leads to enantioselectivity in chiral molecules
Source: Sci Adv. 2025 Oct 29;11(44):eadv5220. doi: 10.1126/sciadv.adv5220 (PMC12571066; doi:10.1126/sciadv.adv5220)
Supplement: Supplementary file 1 — Supplementary Text Figs. S1 to S6 Table S1 References [file sciadv.adv5220_sm.pdf]

Science Advances
AAAS

Supplementary Materials for

Spin polarization driven by molecular vibrations leads to enantioselectivity in chiral molecules

Shinji Miwa *et al.*

Corresponding author: Shinji Miwa, miwa@issp.u-tokyo.ac.jp; Tatsuhiko Ohto, ohto@nagoya-u.jp

Sci. Adv. **11**, eadv5220 (2025)
DOI: 10.1126/sciadv.adv5220

This PDF file includes:

Supplementary Text
Figs. S1 to S6
Table S1
References

Supplementary Text

Chirality dependence on the MC effect

Figure 2A of the main text shows that when the MC effects of 0.25 mM (*S*)-CSA and (*R*)-CSA are compared using CoPt/Au (1 nm), there appears to be a sign inversion and a difference in amplitude. Figure S4 shows the MC effects of 4 mM (*S*)-CSA, (*R*)-CSA, and *rac*-CSA using CoPt/Au electrodes in the Au 0 nm limit. Unlike Fig. 2A of the main text, which shows the first antiparallel peak of the interlayer exchange coupling (at Au 1 nm), the data at Au 0 nm show a complete sign reversal. The data at Au 0 nm represent the first parallel peak of the interlayer exchange coupling.

Additionally, the MC effect of (*R*)-CSA was found to be larger than that of (*S*)-CSA, the saturation of the MC effect was weaker for (*R*)-CSA than for (*S*)-CSA, and that *rac*-CSA did not lie exactly between (*S*)-CSA and (*R*)-CSA. The difference in saturation behavior between the *S*- and *R*-enantiomers may be due to impurities introduced during respective purification processes. Focusing on the initial rise region of the MC effect (< 100 s), the racemic mixture falls between the *S*- and *R*-enantiomers in magnitude. Notably, the MC effect of the *R*-enantiomer remains larger than that of the *S*-enantiomer, and the racemic mixture exhibits a finite response. These observations suggest an intrinsic difference in the magnitude of the MC effect between the *S*- and *R*-enantiomers. In the case of free-standing molecules, reversing molecular chirality results in a complete inversion of the system. However, for interfacial molecules, chirality inversion is expected to substantially alter, but not entirely reverse, the system's asymmetry. This could explain why the magnitude of the MC effects for *S*- and *R*-enantiomers are not the same.

Magnitude of the chirality-induced interlayer exchange coupling driven by molecular vibrations

The chirality-induced exchange coupling originates from the emergence of spin density due to molecular vibrations, as well as from the asymmetry in the oscillation of spin density under an external magnetic field. The former depends primarily on the electron-vibration coupling of the vibrational modes, while the latter depends on spin-vibration coupling, i.e., the interaction between phonons and magnetic moments. A larger value of either parameter is expected to lead to a stronger effective field. For instance, the vibration of the ketone group is a major contributor to the strong electron-vibration coupling in the CSA molecule used in this study. The strong electron-vibration coupling observed in the ketone group is generally attributed to the significant modulation of the C=O bond's electric dipole moment resulting from the large difference in electron negativity between the carbon and oxygen atoms.

In fact, quantitatively predicting the spin density in the chiral molecules remains challenge within current theoretical frameworks. As discussed above, spin-density oscillations induced by molecular vibrations are expected to become asymmetric under an external magnetic field due to spin-vibration coupling. If the asymmetry of the spin-density oscillation can be characterized quantitatively, then the effective field can also be evaluated quantitatively. Regarding spin-vibration coupling, previous research has reported large phonon magnetic moments (see Ref. 67 for example). However, these magnitudes cannot be explained solely by the rotational motion of atomic nuclei. It has been suggested that interactions between atomic nuclei and surrounding conduction electron spins play a crucial role. Even in inorganic crystals, the strength of spin-vibration coupling remains is still unclear. For interfacial organic molecules, such as those considered in this study, conducting a rigorous theoretical analysis is more challenging still. To the best of our knowledge, the quantification of the coupling strength between molecular vibrations

and magnetic moments has not yet been addressed, making it a significant and novel subject for future research.

Length scale of the magnetic interaction in CISS

In this study, the thickness of the Au spacer layer between the CoPt ferromagnetic metal and the CSA chiral molecules appears to correspond to the characteristic length scale of the magnetic interaction relevant to the CISS. However, at high CSA concentrations (> 8 mM), the solution easily penetrates the Au layer, enabling direct interaction with the underlying CoPt, as shown in Fig. S2. Under these conditions, the thickness of the Au layer loses its physical significance and mainly serves to slightly delay the dissolution of CoPt. Previous CISS studies using electrolytes *(8, 16)* commonly employed structures such as Ni/Au (5-10 nm). In those cases, the Au layer provided partial protection to the Ni, however, the Au thickness did not necessarily reflect the intrinsic interaction length scale associated with CISS. With this in mind, we aimed to construct an optimized, though not perfect, experimental system. We used a low CSA concentration (0.25 mM) and selected CoPt, a ferromagnet that is more corrosion-resistant than Ni or Co. To the best of our knowledge, this is the first experimental report in which the Au spacer thickness reliably corresponds to the length scale of the underlying interaction. As shown in Fig. 2D of the main text, the magnetic interaction decays significantly within a few nanometers, strongly suggesting the involvement of interlayer exchange coupling.

Accordingly, it is essential to critically assess whether the nominal spacer thickness between the ferromagnet and the chiral molecules truly represents the intrinsic interaction length scale in the device. In the previous studies where a CISS-induced effective field was observed *(9, 30)*, the effective thickness was likely influenced by specific chemical treatments used during peptide functionalization. For example, in Ref. 30, a relatively thick multilayer structure of MgO(1.5 nm)/Ta(2 nm)/Au(3 nm) was used as a spacer layer between CoFeB and peptide. However, if chemical treatments during peptide adsorption significantly reduce the effective spacer thickness, then the systems in Ref. 30 and the present study may be probing an effective field driven by vibration-driven exchange coupling. Ref. 30 represents the first reported case of detecting this effective field through its influence on skyrmion motion. Meanwhile, CISS-induced magnetization reversal *(9)* is widely acknowledged as an exceptionally large effect. While the effective field proposed in this study may contribute to this phenomenon, an additional amplification mechanism is likely required to fully explain the observed results. One plausible amplification mechanism involves energy dissipation during the chemisorption of peptides onto the Au surface, where a covalent bond is formed. This energy dissipation could explain the strong magnetic interaction observed between the magnetic material and the chiral molecule.

Temperature dependence of the vibration-driven spin polarization

First, we confirm a linear relationship between the induced spin density and the vibrational amplitude in our calculation. Spin polarization is defined as shown in Fig. S6. It is obtained by integrating the spin density over the region indicated by the square in the inset. Taking the vibrational amplitude as the displacement of the C=O bond, we calculate the corresponding coefficient to be $1.9\times10^{-2}\mu_B$ Å^{-1}. Next, we compute the C=O amplitude as a function of temperature and estimate the resulting spin polarization, as shown in Fig. S6. As temperature increases from absolute zero, the amplitude of molecular vibrations increases, leading to a monotonic increase in the induced spin polarization. Since the vibrational mode under

consideration has an energy of 1770 cm^{-1} (219 meV), the spin polarization does not saturate up to room temperature (26 meV), as shown in Fig. S6.

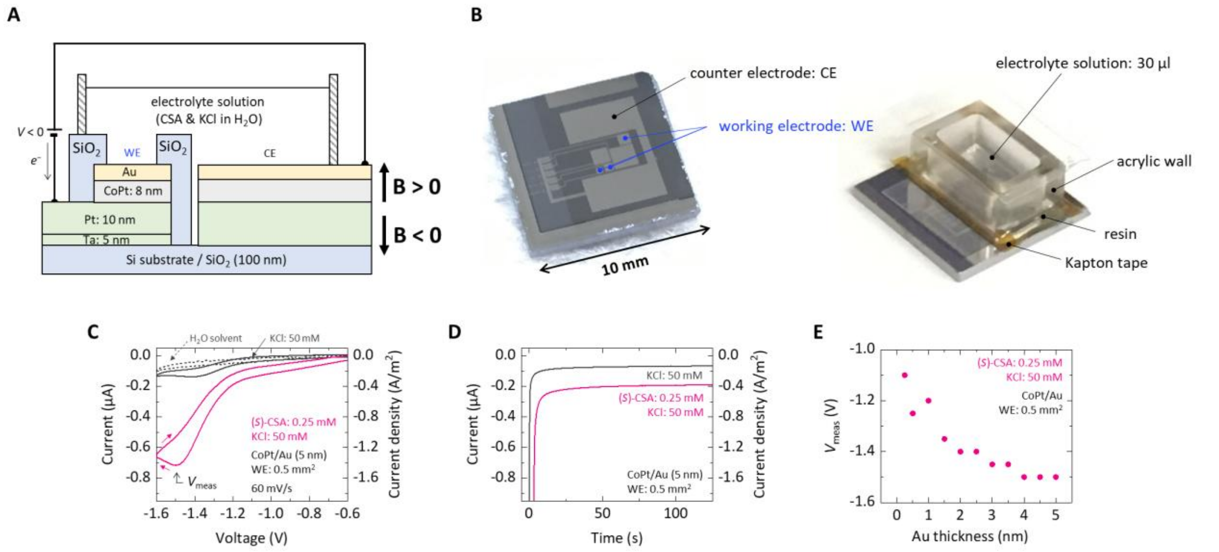

Fig. S1.

Overview of a custom-made electrochemical cell. (**A**) A schematic illustration of the electrochemical cell. (**B**) Photographic images of the electrochemical cell. (**C**) Typical current–voltage characteristics results. (**D**) Typical chronoamperometry results performed under a constant voltage (V_{meas}). (**E**) Voltage (V_{meas}) for chronoamperometry as a function of Au spacer thickness.

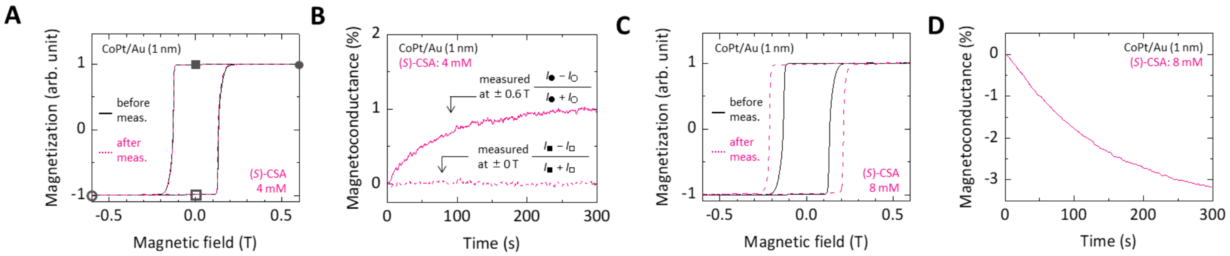

Fig. S2.

Magnetoconductance in devices with varying CSA concentrations. (**A**) Magnetization hysteresis curves of the ferromagnetic CoPt electrode, observed before and after electrical measurements were taken using an electrochemical cell containing (*S*)-CSA at 4 mM and KCl at 200 mM, with a magnetic field applied perpendicular to the film plane. (**B**) Magnetoconductance (MC) effects observed in a CoPt/Au(1 nm) electrode both with (○, ●) and without (■, □) a magnetic field of ±0.6 T during measurements. In both cases, the magnetization of CoPt was polarized as shown in Fig. S2A. (**C**) Magnetization hysteresis curves of the ferromagnetic CoPt electrode, observed before and after electrical measurements with an electrochemical cell containing (*S*)-CSA at 8 mM and KCl at 400 mM. (**D**) MC effect observed in a CoPt/Au(1 nm) electrode with a magnetic field of ±0.6 T during measurements.

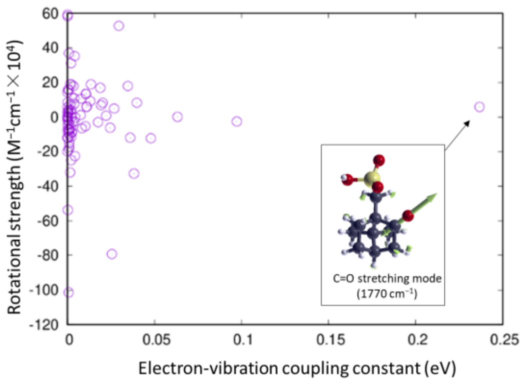

Fig. S3.

Molecular vibration in CSA. This plot shows 87 (31×3−6) vibrational modes according to the electron–vibration coupling constant and rotational strength in the vibrational circular dichroism (VCD) analysis. The C=O stretching mode (1770 cm^{-1}) is displayed in the inset.

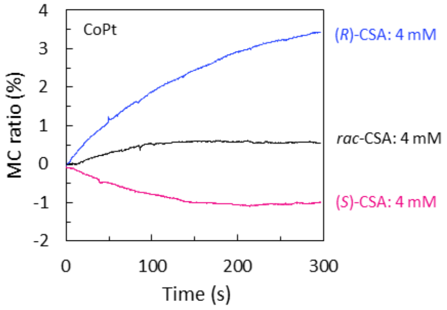

Fig. S4.

MC effect using CoPt electrode. The chirality dependence of the MC effect was studied in CoPt/Au (0 nm) electrodes using electrolyte solution consisting of 4 mM CSA and 200 mM KCl.

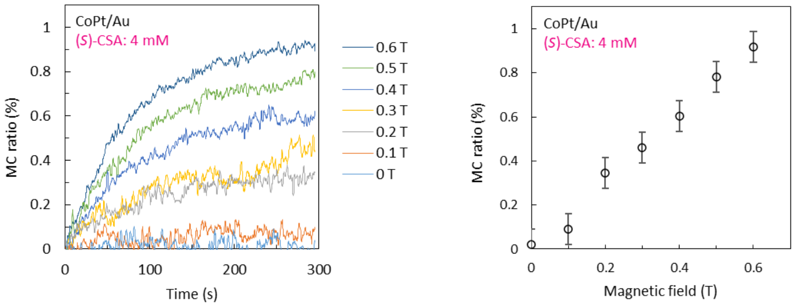

Fig. S5.

Magnetic field dependence of the MC effect. The MC ratio as a function of the external magnetic field in CoPt/Au electrodes using (*S*)-CSA (4 mM).

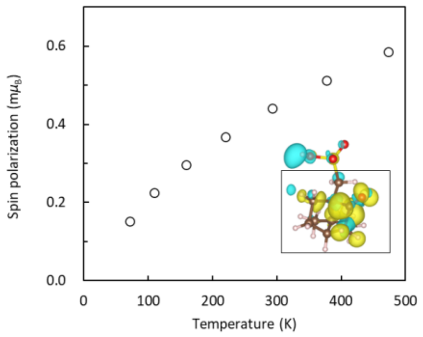

Fig. S6.

Temperature dependence of vibration-driven spin polarization. The calculated spin polarization of (*S*)-CSA is shown as a function of temperature. The vertical axis represents the spin polarization, obtained by integrating the spin density over the region indicated by the square in the inset.

Table S1.
Molecular vibration in CSA.

	Vibration frequency	Electron–vibration coupling	Rotational strength in VCD analysis	Spin–vibration coupling
	(cm^{-1})	(eV)	($M^{-1}cm^{-1}\times10^{4}$)	(bohr per ~0.1 Å displacement)
1	3690	0.0003	−6.88	0.00104
2	3069	0.0000	−0.30	0.00039
3	3060	0.0000	5.53	0.00096
4	3058	0.0001	−11.92	0.00161
5	3056	0.0004	1.79	0.00112
6	3039	0.0000	14.31	0.00068
7	3038	0.0002	−53.76	0.00034
8	3035	0.0001	58.24	0.00085
9	3031	0.0000	−8.18	0.00409
10	3026	0.0008	−7.46	0.00124
11	3007	0.0002	0.16	0.00033
12	3001	0.0004	15.74	0.00086
13	2999	0.0045	−0.28	0.00218
14	2997	0.0011	−6.98	0.00089
15	2975	0.0005	2.52	0.00133
16	2971	0.0000	0.53	0.00305
17	1770	0.2367	5.85	0.00066
18	1474	0.0005	−2.16	0.00097
19	1467	0.0002	8.64	0.00153
20	1460	0.0006	3.21	0.00252
21	1450	0.0000	3.02	0.00037
22	1439	0.0000	−1.75	0.00163
23	1432	0.0003	−0.61	0.00121
24	1403	0.0040	−2.70	0.00153
25	1382	0.0009	−3.61	0.00068
26	1374	0.0176	−2.99	0.00149
27	1353	0.0030	−2.91	0.00062
28	1333	0.0041	35.23	0.00080

29	1296	0.0131	9.23	0.00152
30	1288	0.0008	−9.53	0.00135
31	1275	0.0008	−18.74	0.00107
32	1257	0.0024	11.49	0.00073
33	1247	0.0018	3.53	0.00016
34	1239	0.0039	10.85	0.00162
35	1223	0.0202	8.20	0.00142
36	1202	0.0039	7.61	0.00104
37	1195	0.0078	10.63	0.00091
38	1186	0.0017	7.54	0.00161
39	1160	0.0011	−7.68	0.00170
40	1141	0.0022	18.76	0.00134
41	1132	0.0007	−101.44	0.00093
42	1107	0.0020	−6.25	0.00103
43	1086	0.0013	−10.92	0.00093
44	1071	0.0001	59.20	0.00183
45	1048	0.0022	2.03	0.00124
46	1027	0.0043	−22.55	0.00245
47	1009	0.0002	−12.02	0.00052
48	987	0.0000	−19.92	0.00046
49	969	0.0002	−4.69	0.00189
50	957	0.0008	36.93	0.00094
51	934	0.0006	4.28	0.00153
52	928	0.0007	15.78	0.00150
53	917	0.0021	4.22	0.00189
54	892	0.0106	13.49	0.00097
55	870	0.0025	-8.96	0.00148
56	844	0.0024	0.22	0.00191
57	801	0.0188	16.87	0.00150
58	766	0.0398	8.25	0.00151
59	745	0.0015	−32.02	0.00065
60	704	0.0382	−32.66	0.00310
61	692	0.0021	−24.92	0.00051

62	672	0.0111	5.97	0.00145
63	606	0.001	−14.93	0.00069
64	571	0.0268	4.90	0.00104
65	554	0.0103	−5.30	0.00114
66	548	0.0295	52.72	0.00150
67	516	0.0360	−11.97	0.00179
68	487	0.0245	−6.18	0.00165
69	456	0.0010	−17.16	0.00124
70	423	0.0026	−8.57	0.00113
71	413	0.0109	5.90	0.00066
72	387	0.0075	1.11	0.00170
73	381	0.0022	−11.42	0.00058
74	332	0.0034	17.94	0.00167
75	298	0.0222	0.82	0.00064
76	290	0.0106	13.29	0.00065
77	276	0.0015	19.07	0.00093
78	252	0.0000	−11.89	0.00008
79	236	0.0020	31.10	0.00224
80	229	0.0347	17.97	0.00114
81	202	0.0135	18.89	0.00062
82	175	0.0255	−79.23	0.00024
83	167	0.0083	−6.56	0.00071
84	160	0.0193	6.86	0.00109
85	156	0.0632	0.12	0.00032
86	130	0.0479	−12.29	0.00108
87	122	0.0973	−2.59	0.00146

REFERENCES AND NOTES

1. L. Pasteur, On the relationships between the crystalline form, chemical composition and the direction of optical rotation. *Ann. Chim. Phys.* **24**, 442–459 (1848).

2. M. Gardner, *The Ambidextrous Universe: Mirror Asymmetry and Time-Reversal Worlds* (Penguin Books, 1964).

3. K. Ray, S. P. Ananthavel, D. H. Waldeck, R. Naaman, Asymmetric scattering of polarized electrons by organized organic films of chiral molecules. *Science* **283**, 814–816 (1999).

4. B. Göhler, V. Hamelbeck, T. Z. Markus, M. Kettner, G. F. Hanne, Z. Vager, R. Naaman, H. Zacharias, Spin selectivity in electron transmission through self-assembled monolayers of double-stranded DNA. *Science* **331**, 894–897 (2011).

5. Z. Xie, T. Z. Markus, S. R. Cohen, Z. Vager, R. Gutierrez, R. Naaman, Spin specific electron conduction through DNA oligomers. *Nano Lett.* **11**, 4652–4655 (2011).

6. O. B. Dor, S. Yochelis, S. P. Mathew, R. Naaman, Y. Paltiel, A chiral-based magnetic memory device without a permanent magnet. *Nat. Commun.* **4**, 2256 (2013).

7. E. Medina, L. A. González-Arraga, D. Finkelstein-Shapiro, B. Berche, V. Mujica, Continuum model for chiral induced spin selectivity in helical molecules. *J. Chem. Phys.* **142**, 194308 (2015).

8. P. C. Mondal, C. Fontanesi, D. H. Waldeck, R. Naaman, Spin-dependent transport through chiral molecules studied by spin-dependent electrochemistry. *Acc. Chem. Res.* **49**, 2560–2568 (2016).

9. O. B. Dor, S. Yochelis, A. Radko, K. Vankayala, E. Capua, A. Capua, S.-H. Yang, L. T. Baczewski, S. S. P. Parkin, R. Naaman, Y. Paltiel, Magnetization switching in ferromagnets by adsorbed chiral molecules without current or external magneric field. *Nat. Commun.* **8**, 14567 (2017).

10. K. Banerjee-Ghosh, O. B. Dor, F. Tassinari, E. Capua, S. Yochelis, A. Capua, S.-H. Yang, S. S. P. Parkin, S. Sarkar, L. Kronik, L. T. Baczewski, R. Naaman, Y. Paltiel, Separation of enantiomers by their enantiospecific interaction with achiral magnetic substrates. *Science* **360**, 1331–1334 (2018).

11. H. Lu, J. Wang, C. Xiao, X. Pan, X. Chen, R. Brunecky, J. J. Berry, K. Zhu, M. C. Beard, Z. V. Vardeny, Spin-dependent charge transport through 2D chiral hybrid lead-iodide perovskites. *Sci. Adv.* **5**, eaay0571 (2019).

12. S. Dalum, P. Hedegård, Theory of chiral induced spin selectivity. *Nano Lett.* **19**, 5253–5259 (2019).

13. R. Naaman, Y. Paltiel, D. H. Waldeck, Chiral molecules and the electron spin. *Nat. Rev. Chem.* **3**, 250–260 (2019).

14. S. Miwa, K. Kondou, S. Sakamoto, A. Nihonyanagi, F. Araoka, Y. Otani, D. Miyajima, Chirality-induced effective magnetic field in a phthalocyanine molecule. *Appl. Phys. Express* **13**, 113001 (2020).

15. N. Sukenik, F. Tassinari, S. Yachelis, O. Milo, L. T. Baczewski, Y. Paltial, Correlation between ferromagnetic layer easy axis and the tilt angle of self assembled chiral molecules. *Molecules* **25**, 6036 (2020).

16. T. S. Metzger, S. Mishra, B. P. Bloom, N. Goren, A. Neubauer, G. Shmul, J. Wei, S. Yochelis, F. Tassinari, C. Fontanesi, D. H. Waldeck, Y. Paltiel, R. Naaman, The electron spin as a chiral reagent. *Angew. Chem. Int. Ed.* **59**, 1653–1658 (2020).

17. R. Naaman, Y. Paltiel, D. H. Waldeck, Chiral molecules and the spin selectivity effect. *J. Phys. Chem. Lett.* **11**, 3660–3666 (2020).

18. C. Kulkarni, A. K. Mondal, T. K. Das, G. Grinbom, F. Tassinari, M. F. J. Mabesoone, E. W. Meijer, R. Naaman, Highly efficient and tunable filtering of electrons' spin by supramolecular chirality of nanofiber-based materials. *Adv. Mater.* **32**, e1904965 (2020).

19. Y.-H. Kim, Y. Zhai, H. Lu, X. Pan, C. Xiao, E. A. Gaulding, S. P. Harvey, J. J. Berry, Z. V. Vardeny, J. M. Luther, M. C. Beard, Chiral-induced spin selectivity enables a room-temperature spin light-emitting diode. *Science* **371**, 1129–1133 (2021).

20. P.-H. Huang, K. Taniguchi, M. Shigefuji, T. Kobayashi, M. Matsubara, T. Sasagawa, H. Sato, H. Miyasaka, Chirality-dependent circular photogalvanic effect in enantiomorphic 2D organic-inorganic hybrid perovskites. *Adv. Mater.* **33**, e2008611 (2021).

21. N. Goren, S. Yochelis, G. Jung, Y. Paltiel, Magnetic passivation using chiral molecules. *Appl. Phys. Lett.* **118**, 172401 (2021).

22. K. Kondou, M. Shiga, S. Sakamoto, H. Inuzuka, A. Nihonyanagi, F. Araoka, M. Kobayashi, S. Miwa, D. Miyajima, Y. Otani, Chirality-induced magnetoresistance due to thermally driven spin polarization. *J. Am. Chem. Soc.* **144**, 7302–7307 (2022).

23. K. Kondou, S. Miwa, D. Miyajima, Spontaneous spin selectivity in chiral molecules at the interface. *J. Magn. Magn. Mater.* **585**, 171157 (2023).

24. H. J. Eckvahl, N. A. Tcyrulnikov, A. Chiesa, J. M. Bradley, R. M. Young, S. Carretta, M. D. Krzyaniak, M. R. Wasielewski, Direct observation of chirality-induced spin selectivity in electron donor-acceptor molecules. *Science* **382**, 197–201 (2023).

25. R. Nakajima, D. Hirobe, G. Kawaguchi, Y. Nabei, T. Sato, T. Narushima, H. Okamoto, H. M. Yamamoto, Giant spin polarization and a pair of antiparallel spins in a chiral superconductor. *Nature* **613**, 479–484 (2023).

26. Z. Bian, Y. Nakano, K. Miyata, I. Oya, M. Nobuoka, Y. Tsutsui, S. Seki, M. Suda, Chiral van der Waals superlattices for enhanced spin-selective transport and spin-dependent electrocatalytic performance. *Adv. Mater.* **35**, e2306061 (2023).

27. H. Aizawa, T. Sato, S. Maki-Yonekura, K. Yonekura, K. Takaba, T. Hamaguchi, T. Minato, H. M. Yamamoto, Enantioselectivity of discretized helical supramolecule consisting of achiral cobalt phthalocyanines via chiral-induced spin selectivity effect. *Nat. Commun.* **14**, 4530 (2023).

28. M. R. Safari, F. Matthes, V. Caciuc, N. Atodiresei, C. M. Schneider, K.-H. Ernst, D. E. Bürgler, Enantioselective adsorption on magnetic surfaces. *Adv. Mater.* **36**, 2308666 (2023).

29. I. Abdelwahab, D. Kumar, T. Bian, H. Zheng, H. Gao, F. Hu, A. McClelland, K. Leng, W. L. Wilson, J. Yin, H. Yang, K. P. Loh, Two-dimensional chiral perovskites with large spin Hall angle and collinear spin Hall conductivity. *Science* **385**, 311–317 (2024).

30. Y. Kapton, F. Kammerbauer, T. Balland, S. Yochelis, M. Kläui, Y. Paltiel, Effects of chiral polypeptides on skyrmion stability and dynamics. *Nano Lett.* **25**, 306–312 (2025).

31. L. D. Barron, True and false chirality and absolute asymmetric synthesis. *J. Am. Chem. Soc.* **108**, 5539–5542 (1986).

32. J. Kishine, H. Kusunose, H. M. Yamamoto, On the definition of chirality and enantioselective fields. *Isr. J. Chem.* **62**, e202200049 (2022).

33. G. L. J. A. Rikken, J. Fölling, Electrical magnetochiral anisotropy. *Phys. Rev. Lett.* **87**, 236602 (2001).

34. X. Yang, C. H. van der Wal, B. J. van Wees, Spin-dependent electron transmission model for chiral molecules in mesoscopic devices. *Phys. Rev. B* **99**, 024418 (2019).

35. G. L. J. A. Rikken, N. Avarvari, Comparing electrical magnetochiral anisotropy and chirality-induced spin selectivity. *J. Phys. Chem. Lett.* **14**, 9727–9731 (2023).

36. S. S. P. Parkin, C. Kaiser, A. Panchula, P. M. Rice, B. Hughes, M. Samant, S.-H. Yang, Giant tunnelling magnetoresistance at room temperature with MgO (100) tunnel barriers. *Nat. Mater.* **3**, 862–867 (2004).

37. S. Yuasa, T. Nagahama, A. Fukushima, Y. Suzuki, K. Ando, Giant room-temperature magnetoresistance in single-crystal Fe/MgO/Fe magnetic tunnel junctions. *Nat. Mater.* **3**, 868–871 (2004).

38. D. D. Djayaprawira, K. Tsunekawa, M. Nagai, H. Maehara, S. Yamagata, N. Watanabe, S. Yuasa, Y. Suzukik, K. Ando, 230% Room-temperature magnetoresistance in CoFeB/MgO/CoFeB magnetic tunnel junctions. *Appl. Phys. Lett.* **86**, 092502 (2005).

39. I. Meirzada, N. Sukenik, G. Haim, S. Yochelis, L. T. Baczewski, Y. Paltiel, N. Bar-Gill, Long-time-scale magnetization ordering induced by an adsorbed chiral monolayer on ferromagnets. *ACS Nano* **15**, 5574–5579 (2021).

40. F. J. Jedema, A. T. Filip, B. J. van Wees, Electrical spin injection and accumulation at room temperature in an all-metal mesoscopic spin valve. *Nature* **410**, 345–348 (2001).

41. P. Grünberg, R. Schreiber, Y. Pang, M. B. Brodsky, H. Sowers, Layered magnetic structures: Evidence for antiferromagnetic coupling of Fe layers across Cr interlayers. *Phys. Rev. Lett.* **57**, 2442–2445 (1986).

42. J. Fransson, Charge redistribution and spin polarization driven by correlation induced electron exchange in chiral molecules. *Nano Lett.* **21**, 3026–3032 (2021).

43. T. K. Das, F. Tassinari, R. Naaman, J. Fransson, Temperature-dependent chiral-induced spin selectivity effect: Experiments and theory. *J. Phys. Chem. C* **126**, 3257–3264 (2022).

44. J. Fransson, Chirality-induced spin selectivity: The role of electron correlations. *J. Phys. Chem. Lett.* **10**, 7126–7132 (2019).

45. J. Fransson, Vibrational origin of exchange splitting and chiral-induced spin selectivity. *Phys. Rev. B* **102**, 235416 (2020).

46. C. Vittmann, J. Lim, D. Tamascelli, S. F. Huelga, M. B. Plenio, Spin-dependent momentum conservation of electron-phonon scattering in chirality-induced spin selectivity. *J. Phys. Chem. Lett.* **14**, 340–346 (2023).

47. M. Julliere, Tunneling between ferromagnetic films. *Phys. Lett. A* **54**, 225–226 (1975).

48. F. G. Cottrell, Der reststrom bei galvanischer polarisation, betrachtet als ein dffusionsproblem. *Z. Phys. Chem.* **42**, 385–431 (1903).

49. A. Fuβ, S. Demokritov, P. Grünberg, W. Zinn, Short- and long period oscillations in the exchange coupling of Fe across epitaxially grown Al- and Au-interlayers. *J. Magn. Magn. Mater.* **103**, L221–L227 (1992).

50. P. Bruno, C. Chappert, Ruderman-Kittel theory of oscillatory interlayer exchange coupling. *Phys. Rev. B* **46**, 261–270 (1992).

51. M. A. Ruderman, C. Kittel, Indirect exchange coupling of nuclear magnetic moments by conduction electrons. *Phys. Rev.* **96**, 99–102 (1954).

52. T. A. Kasuya, A theory of metallic ferro- and antiferromagnetism on Zener's model. *Prog. Theor. Phys.* **16**, 45–57 (1956).

53. K. Yosida, Magnetic properties of Cu-Mn alloys. *Phys. Rev.* **106**, 893–898 (1957).

54. A. Rettori, L. Trallori, P. Politi, M. G. Pini, M. Macciò, Surface magnetic reconstruction. *J. Magn. Magn. Mater.* **140–144**, 639–642 (1995).

55. G. Schaack, Magnetic-field dependent phonon states in paramagnetic CeF_3. *Solid State Commun.* **17**, 505–509 (1975).

56. L. Zhang, Q. Niu, Angular momentum of phonons and the Einstein-de Haas effect. *Phys. Rev. Lett.* **112**, 085503 (2014).

57. S. Alwan, Y. Dubi, Spinterface origin for the chirality-induced spin-selectivity effect. *J. Am. Chem. Soc.* **143**, 14235–14241 (2021).

58. I. Carmeli, G. Leitus, R. Naaman, S. Reich, Z. Vager, Magnetism induced by the organization of self-assembled monolayers. *J. Chem. Phys.* **118**, 10372–10375 (2003).

59. K. Yakushiji, H. Kubota, A. Fukushima, S. Yuasa, Perpendicular magnetic tunnel junctions with strong antiferromagnetic interlayer exchange coupling at first oscillation peak. *Appl. Phys. Express* **8**, 083003 (2015).

60. G. Kresse, J. Hafner, Norm-conserving and ultrasoft pseudopotentials for first-row and transition elements. *J. Phys. Condens. Matter.* **6**, 8245 (1994).

61. P. E. Blöchl, Projector augmented-wave method. *Phys. Rev. B* **50**, 17953–17979 (1994).

62. J. P. Pedrew, K. Burke, M. Ernzerhof, Generalized gradient approximation made simple. *Phys. Rev. Lett.* **77**, 3865–3868 (1996).

63. J. M. Soler, The SIESTA method for *ab initio* order-*N* materials simulation. *J. Phys. Condens. Matter.* **14**, 2745 (2002).

64. T. Ohto, I. Rungger, K. Yamashita, H. Nakamura, S. Sanvito, Ab initio theory for current-induced molecular switching: Melamine on Cu(001). *Phys. Rev. B* **87**, 205439 (2013).

65. M. J. Frisch, G. W. Trucks, H. B. Schlegel, G. E. Scuseria, M. A. Robb, J. R. Cheeseman, G. Scalmani, V. Barone, G. A. Petersson, H. Nakatsuji, X. Li, M. Caricato, A. V. Marenich, J. Bloino, B. G. Janesko, R. Gomperts, B. Mennucci, H. P. Hratchian, J. V. Ortiz, A. F. Izmaylov, J. L. Sonnenberg, D. Williams-Young, F. Ding, F. Lipparini, F. Egidi, J. Goings, B. Peng, A. Petrone, T. Henderson, D. Ranasinghe, V. G. Zakrzewski, J. Gao, N. Rega, G. Zheng, W. Liang, M. Hada, M. Ehara, K. Toyota, R. Fukuda, J. Hasegawa, M. Ishida, T. Nakajima, Y. Honda, O. Kitao, H. Nakai, T. Vreven, K. Throssell, J. A. Montgomery Jr., J. E. Peralta, F. Ogliaro, M. J. Bearpark, J. J. Heyd, E. N. Brothers, K. N. Kudin, V. N. Staroverov, T. A. Keith, R. Kobayashi, J. Normand, K. Raghavachari, A. P. Rendell, J. C. Burant, S. S. Iyengar, J. Tomasi, M. Cossi, J. M. Millam, M. Klene, C. Adamo, R. Cammi, J. W. Ochterski, R. L. Martin, K. Morokuma, O. Farkas, J. B. Foresman, D. J. Fox, Gaussian 16, Revision C.01 (Gaussian Inc., 2016).

66. S. Jähnigen, Vibrational circular dichroism spectroscopy of chiral molecular crystals: Insights from theory. *Angew. Chem. Int. Ed.* **62**, e202303595 (2023).

67. J. Luo, T. Lin, J. Zhang, X. Chen, E. R. Blackert, R. Xu, B. I. Yakobson, H. Zhu, Large effective magnetic fields from chiral phonons in rare-earth halides. *Science* **382**, 698–702 (2023).
